# Modelling and Correcting the Effects of Tremor on Touchscreen Interaction

**DOI:** 10.3390/s26185981

**Published:** 2026-09-21

**Authors:** Mark D. Dunlop, I. Scott MacKenzie

**Affiliations:** 1 Computer & Information Sciences, University of Strathclyde, Glasgow G1 1XH, UK; 2 Electrical Engineering & Computer Science, York University, Toronto, ON M3J 1P3, Canada; mack@yorku.ca

**Keywords:** tremor, hand tremor, induced tremor, touchscreen interaction, ISO 9241-411, Fitts’ law, touch correction

## Abstract

Essential tremor and Parkinson’s tremor are common movement disorders, affecting approximately 7% of adults over 65, increasing with age. Although numerous studies have used keyboard timing, stand-alone sensors, or smartphone sensors to identify and measure tremor severity, relatively little research has isolated and examined how tremor impacts overall mobile interaction and usability models. This study investigated controlled fixed levels of induced tremor in a standardised ISO pointing task to quantify the effect on usability models of different levels of tremor. Tremor was induced using a wired glove delivering 4 Hz stimulation at 0, 15, 19, and 23 mA (approximating the range from slight to marked tremor). The results show that the Fitts’ throughput was 5.43 bps with no induced tremor, with reductions of 9.4%, 20.3%, and 28.9% at the active levels. Fitts’ law models showed high correlations that improved further when applying the Welford correction for tremor, supporting use of the adjustment when tremor is present. The study also indicates that machine-learned correction significantly improves touch accuracy when using smartphone sensors.

## 1. Introduction

Essential tremor (ET) is a chronic condition characterised by involuntary rhythmic tremor, most commonly in the hands and arms. A recent meta-analysis [1] estimated prevalence in those aged 65 years or older at 5.79%, increasing to 9.30% in the oldest age groups (80s, 90s, and older). The International Essential Tremor Foundation estimates that 10 million Americans have ET (https://www.essentialtremor.org/wp-content/uploads/2013/07/FactSheet012013.pdf accessed on 1 September 2026). Tremor is also one of the main symptoms of Parkinson’s Disease (PD), a progressive neurological condition that affects 0.4% of adults between 65 and 74, rising to 1.9% in those over 80 [2]. PD is often diagnosed late; moreover, its symptoms may be present up to 10 years before clinical diagnosis [3]. The Parkinson’s Foundation estimates that 1.1 million people in the U.S. are living with PD (https://www.parkinson.org/understanding-parkinsons/statistics accessed on 1 September 2026). While ET and PD both result in tremor, the nature of this tremor varies between conditions [4] and individuals [5,6].

Several studies report that tremor affects interaction with touchscreens and that smartphone interaction can serve as a reliable diagnostic and assessment tool [7,8,9]. Iakovakis et al. showed that subtle fine-motor impairments in early Parkinson’s can be estimated through natural smartphone touchscreen typing [10]. Studies also note that motion sensors can identify people with tremor and provide an opportunity for “continuous, unobtrusive, objective, and ecologically valid data collection” [11]. However, few studies have investigated how tremor affects models of interaction with mobile devices or whether tremor can be compensated for by using phone sensors alone. Prior work has reported slower movement times under tremor ([7,12]) and encouraging results for correcting errors using a combination of ring-worn and phone-based sensors. Improving both speed and accuracy is key to enhancing user experience for adults living with tremor and, for progressive conditions, to continue quality use for longer. This is important for older adults to maintain access to mobile apps for entertainment, connecting with loved ones, health and well-being applications, and management of financial or utility service accounts that are increasingly mobile-first.

This paper investigates how Fitts’ law models are affected by different levels of induced tremor, using multiple tremor levels for the first time. We also analyse the collected data to investigate the potential for correcting touch locations based on smartphone sensor measurements during tapping. While our work is motivated by supporting people living with tremor, the studies reported here used a Parkinson’s simulation kit to induce tremor in participants without a tremor condition, allowing us to quantify the impact of controlled tremor levels. Our research questions were:

RQ 1. What are the throughput levels, parameter values, and quality of fit with Fitts’ Law for different levels of induced tremor during standardised smartphone touch-screen interaction?
RQ 2. Does the Welford correction for tremor improve fit with Fitts’ Law with different levels of induced tremor?
RQ 3. To what extent can tremor be compensated for using smartphone sensors alone?

## 2. Background

The standard approach to diagnosing Parkinson’s involves clinical observation and judgment [13,14]. This is difficult in the early stages, when symptoms are subtle. As such, computer-based assessment is used to assist clinicians and to monitor tremor-related conditions outside clinical settings. Tremor, bradykinesia (slowness of movement), rigidity, and postural instability are key aspects of diagnosis, with tremor and bradykinesia often combined in detection.

The spiral drawing test is a standard clinical pen-and-paper assessment that involves the patient drawing Archimedean spirals on paper for a clinician to assess stability of movement [13]. Replications on touchscreens show significant differences between patients with Parkinson’s and control groups [8,15] and good assessment performance compared to trained raters [16]. Pressure sensing with a tablet stylus, when combined with coordinate data, can assess subtle motor abnormalities indicating clinical problems in the early stages [17]. In addition to drawing tasks, tapping exercises can differentiate people with and without PD [18,19]. Montague et al. conducted a four-week in-the-wild study with motor-impaired users and a Sudoku app on a mobile touchscreen; they showed that “not only does interaction performance vary significantly between users, but also that an individual’s interaction abilities are significantly different between device sessions” [9] (p. 123).

Regular tapping on keyboards also shows promise for early unobtrusive detection on mobile devices [10,20] and desktop keyboards [2,21,22,23,24,25,26,27] to support out-of-clinic monitoring. Gestural interactions, including gesture typing, can identify early-stage Parkinson’s [28]. Nicolau and Jorge [29] detailed how tremor affects text-entry errors, with users having higher insertion, substitution, and omission errors compared to a control group. Wang et al. [30] confirmed this and developed an elastic probabilistic model of typing errors that led to faster typing with fewer errors when used by people with PD. Using a mobile game, Ling et al. [7] showed slower movement times and higher variance for participants with PD. They also investigated a model fit using the Finger–Fitts model [31] and used a CNN-based model to detect PD.

Stand-alone accelerometers and gyroscopes can also assist clinical diagnosis and provide accurate objective assessments in free-living conditions [32,33,34,35,36]. LeMoyne et al. [37] used a glove-mounted smartphone and showed that it successfully quantified tremor characteristics in public or private settings away from the clinic. Denault et al. [38] conducted studies with participants performing tasks with a smartphone held in their hand and found reliable time- and frequency-domain tremor characteristics for medically relevant tremor assessments, while Woods et al. [4] showed that a smartphone-based application can discriminate between Parkinson’s and essential postural tremors with over 96% accuracy.

After interviews with people with Parkinson’s, Plaumann et al. [12] developed an approach to automatically correct touch inputs based on accelerometer data using a finger-worn sensor paired with smartphone sensors. In an induced-tremor study, they reported up to 40% fewer target misses in user studies when inducing moderate tremor—a result they confirmed in a study conducted with participants with tremor.

Beyond tremor analysis, it has also been shown that personalised correction models can adjust for individual tapping behaviour [39] and, assisted by sensor data, improve the accuracy of tapping while walking [40].

In the research reported here, we were interested in isolating the effect of tremor, without any associated bradykinesia (slowness of movement that commonly presents alongside tremor), and measuring performance using standardised metrics, such as throughput in ISO standard tasks, and modelling the effect of tremor with Fitts’ law for different tremor levels. We also investigated the potential of using on-board accelerometer and gyroscope data to correct for tapping errors in post-study analysis.

## 3. Inducing Hand Tremor

**Figure 1:**
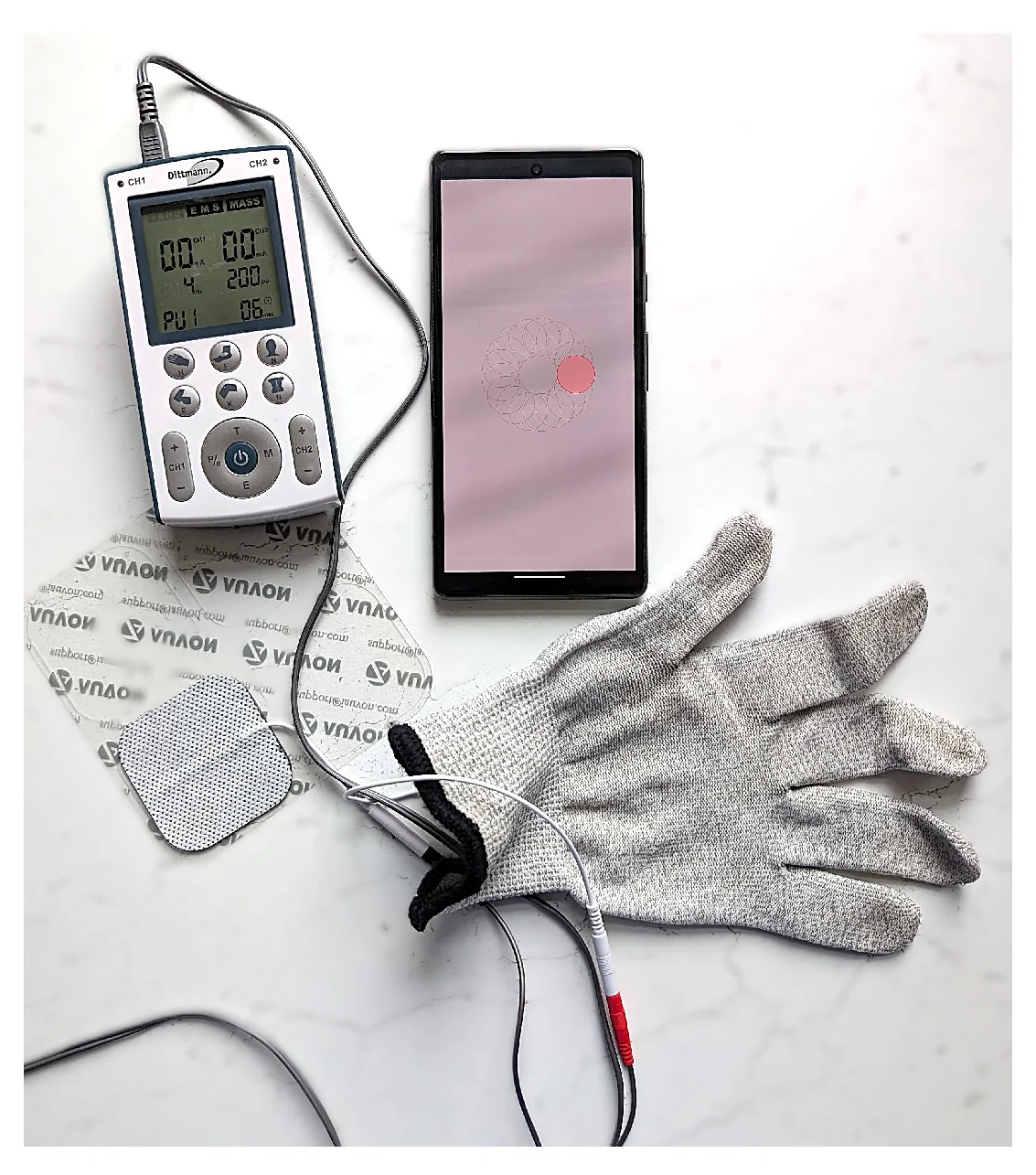
Apparatus: A Dittmann TEN 250 wired to an adhesive electrode and a conductive glove (supplied as part of SD&C Parkinson tremor simulation set from SD&C GmbH, Schweitenkirchen, Germany); a Google Pixel 7a (sourced from Amazon, London, UK) running FittsTouch.

Controlled tremor was induced by a Dittmann Health TEN 250 unit, included with the SD&C GmbH Parkinson Tremor Simulation Set (https://senior-suit.com/product/parkinson-tremor-simulation-set/ accessed on 1 September 2026.) along with gloves and arm electrodes. The Dittmann unit is available over the counter as a three-in-one TENS, ENS and massage unit. It has two output channels and is powered by four 1.5 volt AAA batteries. In our study, medical-grade adhesive electrodes were used along with the supplied conductive glove. See Figure 1.

### Fitts’ Law Testing and ISO 9241-411

**Figure 2:**
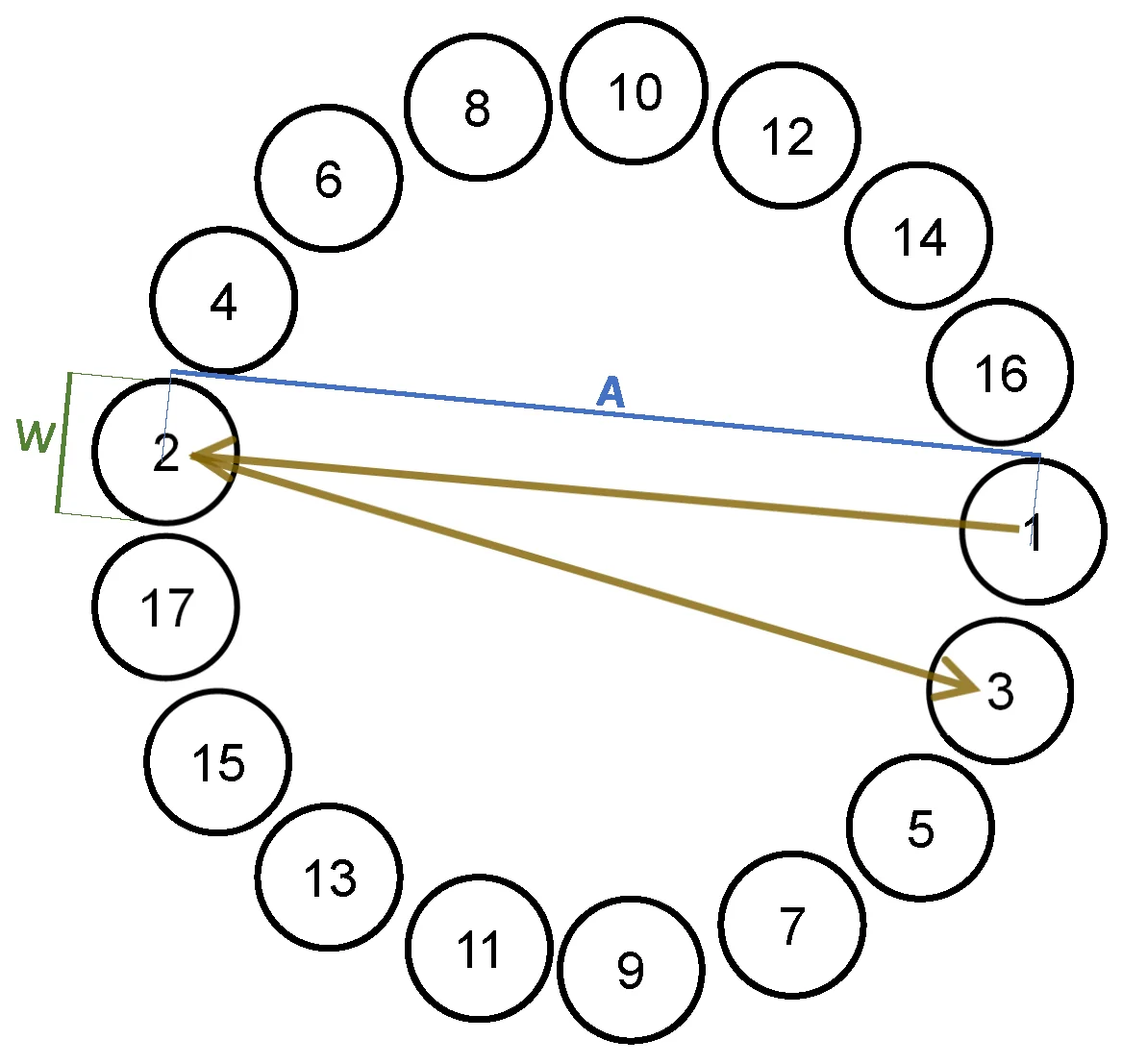
Standard two-dimensional target selection task in ISO 9241-411 [43] showing a sequence of trials with 17 targets. Arrows indicate initial three tap targets with target amplitude *A* and target width *W* as indicated.

Fitts’ law [41] provides a well-established protocol for evaluating target selection on computing systems [42]. This is particularly true since the mid-1990s with the inclusion of Fitts’ law testing in the ISO 9241-411 standard for evaluating non-keyboard input devices [42,43]. The most common ISO evaluation procedure uses a two-dimensional (2D) task with targets of width *W* arranged in a circle (Accurate measurements for throughput require an odd number of targets around the layout circle [44]). Selections proceed in a sequence moving across and around the circle. See Figure 2. Each movement covers an amplitude *A*, the diameter of the layout circle. The movement time (MT, in seconds) is recorded for each trial and averaged over the sequence of trials.

The difficulty of each trial is quantified using an index of difficulty (*ID*, in bits) calculated from *A* and *W* as

(1) $$\mathrm{ID}\;=\;\log_{2}(\frac{A}{W}+1).$$

The main performance measure in ISO 9241-411 is throughput (*TP*, in bits/second or bps), which is calculated over a sequence of trials as the *ID*:*MT* ratio:

(2) $$\mathrm{TP}\;=\;\frac{{\mathrm{ID}}_{e}}{MT}.$$

The standard specifies using the effective index of difficulty (*ID*_e_), which includes an adjustment for accuracy to reflect the spatial variability in responses:

(3) $${\mathrm{ID}}_{e}\;=\;\log_{2}(\frac{A}{W_{e}}+1)$$

with

(4) $$W_{e}\;=\;4.133\times {\mathrm{SD}}_{x}.$$

The term *SD*_x_ is the standard deviation in the selection coordinates computed over a sequence of trials. With the adjustment for accuracy included, throughput is a single measure embedding both the speed ($\frac{1}{MT}$) and accuracy (${\mathrm{SD}}_{x}$) of the user’s performance. We refer the reader to other sources on the background and underlying theory for Equations (3) and (4) [45], [46] (pp. 147–148) and [47].

## 4. Method

As a controlled investigation, data were captured from participants in a laboratory environment using pre-set levels of tremor stimulation as induced via the TENS apparatus described in Section 3.

### 4.1. Participants

Participants were recruited through the University of Strathclyde’s student mailing lists. Several exclusion criteria were applied: people under 18; people with heart disease, cardiac arrhythmia or other heart problems; people with active implants (e.g., pacemakers, spinal cord stimulators, or in-dwelling pumps/monitors, including blood sugar monitors); and additional categories for safety based on the TEN250 manual and guides from Physiopedia [48], Carex [49], and the Association of Paediatric Chartered Physiotherapists [50]. The study was conducted in a local office space under institutional ethics board approval.

A sample size of 24 participants was determined by power analysis (α = 0.05, 1−β = 0.80) to detect a medium-to-large effect (*f* = 0.35) in a repeated-measures ANOVA with four conditions in a within-subjects Latin square design. Of the 24 recruited participants used for analysis, 12 identified as male, 11 as female, and 1 as queer; their ages ranged from 18 to 55 years (mean = 25.2). No participant had contraindications for TENS use, as confirmed in our consent form and verbally. In total, 30 participants were recruited: one participant withdrew for undisclosed contraindication before the study began; five participants took part in the study and were thanked and received compensation, but had their data removed from analysis (3 due to logging errors resulting in partial loss of data and 2 due to uncomfortable excessive induced tremor).

### 4.2. Apparatus

Testing used a Google Pixel 7a smartphone (Android 14) running a version of FittsTouch (https://www.yorku.ca/mack/FittsLawSoftware/ accessed on 30 Jul 2024) that was modified to collect accelerometer and gyroscope data along with touch data and was upgraded to work with Android 11 through 14 (SDK levels 30–34). We recorded accelerometer and gyroscope readings on each keystroke, but sampled at 40 Hz (this approach reduces stored data and allows the code to sleep when inactive, thus saving battery life while avoiding sample aliasing related to the Nyquist rate). In line with the original FittsTouch specification (https://www.yorku.ca/mack/ExperimentSoftware/javadoc/ca/yorku/cse/mack/fittstouch/FittsTouchActivity.html accessed on 30 Jul 2024), two types of error were recorded: (1) misses, where the user tapped near but outside the target, were logged as errors and are reported below; and (2) outliers, where the traversed distance was less than 50% of the specified amplitude, required the user to repeat the sequence of same-sized targets.

Tremor was induced at various stimulation levels using the Dittmann Health TENS unit described in Section 3. Tremor associated with medical conditions can be categorised as resting or action (with action tremor further categorised as postural, isometric, or kinetic). These differ in amplitude and typical frequency, with resting tremor having a low frequency of 3 to 6 Hz and postural a higher range of 4 to 12 Hz [51]. In our study, the TENS unit supplied stimulation at 4 Hz with 200 µs pulses, as recommended by the kit suppliers. This rate is typical of resting tremor and it has also been shown that resting tremor tends to continue during typing [52].

**Figure 3:**
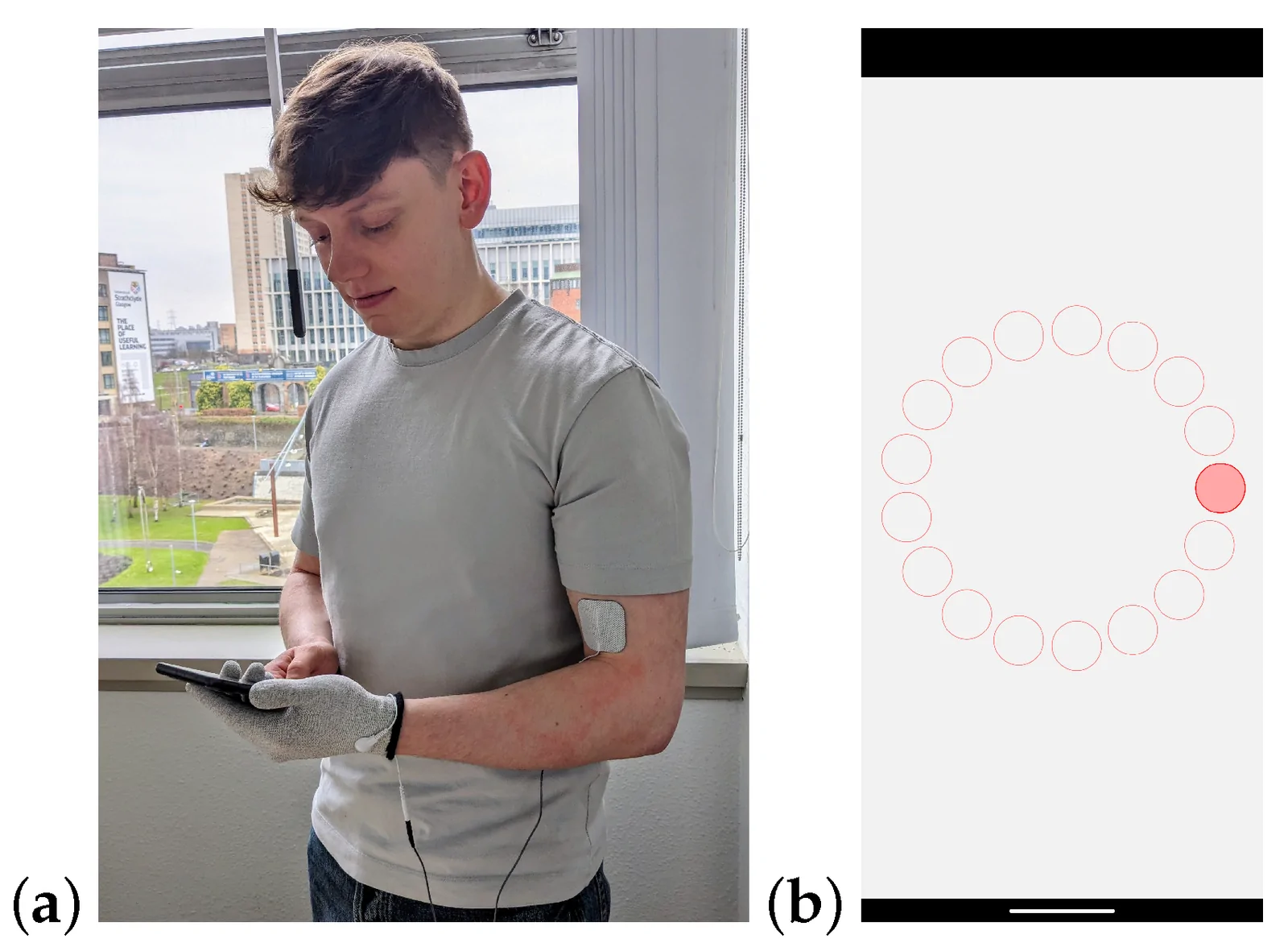
Testing procedure showing (**a**) a participant wearing the tremor stimulation kit and (**b**) the FittsTouch target-selection task interface.

In pre-trial tests, we established that a 15 mA signal induced a mild tremor, similar to level 1 (“slight”) on the Fahn–Tolosa–Marín Clinical Rating Scale for Tremor (FTM) [13]. A setting of 25 mA approximated level 3 (“marked”) on the FTM, but felt slightly too uncomfortable. In the main study, four levels of stimulation were thus used: none (0 mA), light (15 mA), medium (19 mA), and strong (23 mA). Participants stood during the study, tapping targets on the display with a finger of their dominant hand while wearing an electrode glove on their non-dominant hand, with a secondary electrode placed on the same forearm (Figure 3a). We considered instrumenting both hands but opted against it, as tremor is often asynchronous—a state difficult to consistently achieve with TENS units (see Discussion and Limitations).

### 4.3. Procedure

Participants were welcomed to the study and asked to read, or confirm they had read, a participant information sheet (in particular, the exclusion criteria), sign a consent form, and complete a short demographics form. A demonstration of the TENS kit was initially given by the researcher on his own hand and arm and then on the participant’s hand and arm, with a slowly increasing strength of stimulation. The participant then practised using FittsTouch. Practice used no stimulation initially, followed by increasing stimulation using the levels noted above. We stressed to participants that if they felt pain or excessive discomfort, the study would stop immediately. Two excluded participants reported discomfort at the strongest TENS setting; at this point, the study was either stopped or continued without this setting at the participant’s request (all data were excluded from analysis).

Data collection occurred in four short testing phases, with a phase for each stimulation level. Each phase was limited to six minutes for participant comfort. In each phase, the participant performed nine sequences of FittsTouch entry, one for each target amplitude-width condition. Each sequence presented 17 targets in a 2D layout circle, as per Figure 2 and Figure 3b. During the main task phase, participants stood to prevent resting their arm on the table surface. Participants were instructed to hold the phone in their non-dominant hand and tap with their other hand (as seen in Figure 3a). After each phase, participants were allowed to sit, relax, and complete a NASA TLX form [53] before being invited to take a short break. As we were primarily interested in individual TLX dimensions, we omitted the TLX pairing phase to reduce completion time.

At the end of the study, participants were briefly interviewed about their interaction with the system. Handwritten notes were taken to reduce formality.

### 4.4. Design

The study was a 4 × 3 × 3 within-subjects design with the following independent variables and levels:

- Stimulation level (none, light, medium, strong);
- Target amplitude (213, 425, 850 px, range approx. 12–48 mm);
- Target width (89, 133, 213 px, range approx. 5–12 mm).

Each condition presented a sequence of target selection trials with 17 targets positioned around a layout circle. The main independent variable was stimulation level. Target amplitude and target width were included to ensure the testing conditions spanned a reasonable range of task difficulties and to allow building Fitts’ law regression models. Using Equation (1) with the values above, there were nine difficulty conditions with values ranging from *ID* = 1.00 bit to *ID* = 3.41 bits.

The dependent variables were throughput (bps), movement time (ms), and error rate (%). Data were also collected from the testing device’s accelerometer and gyroscope as described above. Responses to the NASA TLX questionnaire were also collected and analysed in addition to verbal comments from participants.

Stimulation level was counterbalanced to offset order effects. Participants were organised into four groups, with the order of stimulation level being different for each group according to a 4 × 4 balanced Latin square. The target amplitude and target width conditions were randomised within stimulation level.

The total number of trials was 14,688 (=24 participants × 4 stimulation levels × 3 target amplitudes × 3 target widths × 17 targets/sequence). Test sessions took about 30 min per participant, including initial practice and form completion. The performance trials took about 17 min on average.

## 5. Results

The group effect on each dependent variable by stimulation level was not significant (*p* > 0.05). Thus, counterbalancing had the desired effect of offsetting performance differences due to the order of testing the stimulation levels.

### 5.1. Throughput

**Figure 4:**
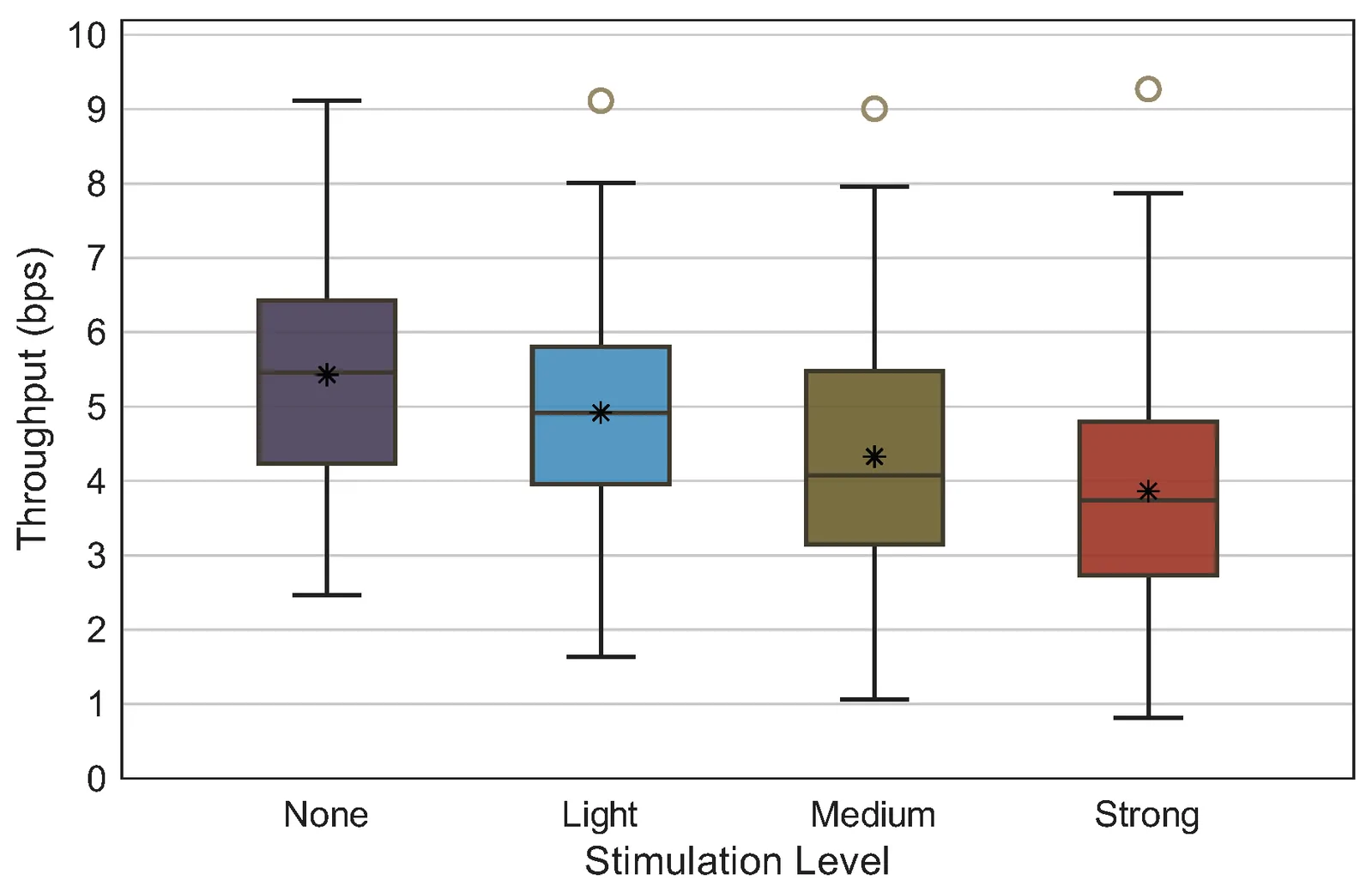
Throughput (bps) by stimulation level. Asterisks show the means.

The grand mean for the throughput was 4.63 bps. By increasing the stimulation level, the means were 5.43 bps (none), 4.92 bps (light), 4.33 bps (medium), and 3.83 bps (strong). The differences were statistically significant (*F*(3, 60) = 27.7, *p* < 0.0001). A post hoc pairwise Scheffé test indicated significant differences (*p* < 0.05) for the none–medium, none–strong, and light–strong pairs. See Figure 4.

The throughput observed with no stimulation is in line with other research using touch-based target selection [54,55,56]. The drop in throughput with increased tremor induced via the TENS apparatus was expected and is clearly seen in Figure 4. A contribution of the present research is to quantify the expected drop in performance for users experiencing hand tremor due to a condition such as Parkinson’s or Essential Tremor. Throughput is particularly useful for this, since the measure embeds speed and accuracy. The observed drops in performance relative to no tremor stimulation were 9.4% (light), 20.3% (medium), and 28.9% (strong).

### 5.2. Movement Time and Error Rate

**Figure 5:**
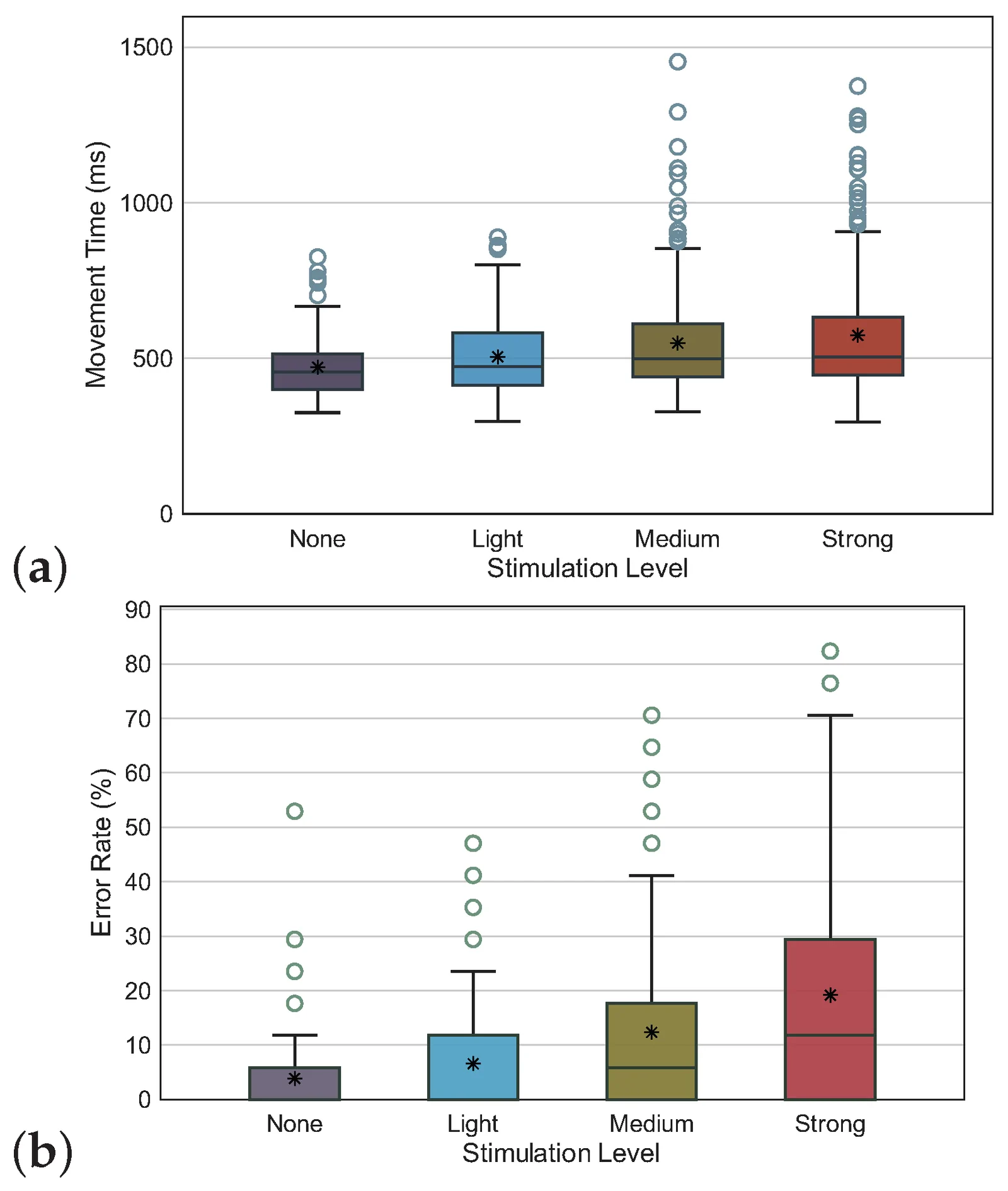
Results for stimulation level by (**a**) movement time (ms) and (**b**) error rate (%). Asterisks show the means.

Although throughput is the only performance measure cited in ISO 9241-411 [43], research in HCI typically includes additional measures for speed and accuracy. Thus, we also present results for movement time (ms) and error rate (%). See Figure 5 for the results by stimulation level. The grand mean for movement time was 525 ms per trial. As expected, movement times increased with greater levels of induced tremor. Compared to no induced tremor (471 ms), the means and percent increases by stimulation level were 504 ms (+7.0%) for light, 549 ms (+16.6%) for medium, and 574 ms (+21.9%) for strong. The differences were statistically significant (*F*(3, 60) = 8.25, *p* < 0.005). The outliers seen in the box plots in Figure 5a for the medium and strong conditions reflect the challenges participants faced while holding the device in their non-dominant hand when subjected to tremor.

The grand mean for error rate was 10.5%. As often occurs, the differences were more erratic for error rate than for movement time. Compared to no induced tremor (3.9%), the means and factor increases for error rate were 6.6% (1.7×) for light, 12.3% (3.2×) for medium, and 19.2% (4.9×) for strong. The differences were statistically significant (*F*(3, 60) = 47.7, *p* < 0.0001). Although outliers appear in the box plots for all stimulation levels (Figure 5b), the number of outlier points is only 15 or 1.7% of the 864 trial sequences in the user study.

### 5.3. Task Load and Qualitative Responses

**Figure 6:**
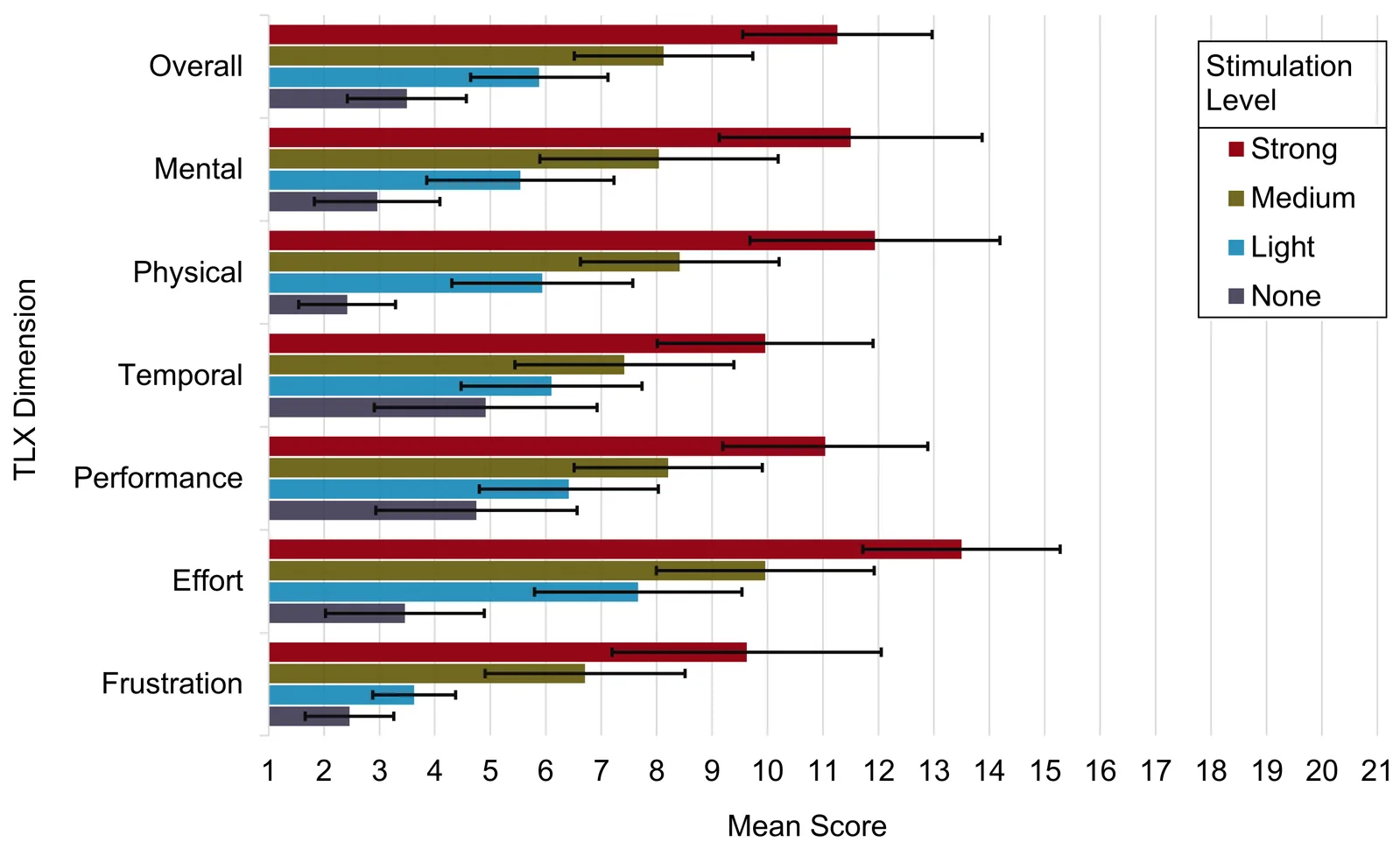
NASA TLX results for overall (plain summation) and six dimensions for four TENS stimulation levels (error bars 95% CI).

All NASA TLX dimensions showed an increase per level of TENS stimulation. See Figure 6. Considering the overall score, calculated as a simple summation, Mauchly’s test of sphericity indicated that the assumption of sphericity was violated ($\chi^{2}$(5) = 39.40, *p* < 0.001) and therefore, a Greenhouse–Geisser correction was used (ε = 0.477). The ANOVA indicated a significant difference in responses by stimulation level (*F*(1.43, 32.9) = 60.83, *p* < 0.001) with means of 3.49 (none), 5.87 (light), 8.13 (medium), and 11.26 (strong). Post hoc paired *t*-tests using a Bonferroni correction (α = 0.0083) indicated that the means of all stimulation-level pairs were significantly different. Although the relative difference between stimulation levels for dimensions was small, an ANOVA also indicated a significant difference in responses by stimulation levels and temporal dimension (Greenhouse–Geisser corrected with ε = 0.733, *F*(2.2, 50.58) = 14.47, *p* < 0.001), thus indicating an increased feeling of time pressure with added TENS stimulation (pairs none–medium, none–strong, light–strong, and medium–strong were significant in post hoc *t*-tests).

The study also included discussion after testing, with comments added to comments made during testing. These confirmed that the stimulation levels were appropriate, with one participant commenting, when moving from strong to medium stimulation, that “this is much easier…definitely a huge difference in mental demand”, while a participant advancing from medium to strong said that it “got a lot more difficult quickly, disproportionally more difficult”, and another simply stated that “hard is really hard”. Time pressure led to a couple of comments, with one participant noting they felt “in a rush to complete the task with this shaky hand”, while another commented “strong was interesting… it didn’t bother me—it forced me to slow down”. No users reported pain, but some excluded users reported excessive tremor and discomfort; others commented that it was a “weird feeling” or that they “quite like the feeling of it” (at low strength). Two users commented on erroneous double taps, with one clarifying that the “tremor moves the phone into my finger”. Although users were encouraged to take a break between conditions, three noted a residual effect, giving them the feeling that their “muscles [were] still not in resting state” or that it “feels like the machine is still on”. Other comments were “once you miss one it gets a bit more frustrating trying to hit the others”, “interesting”, “fun”, and “I got used to the tremor”.

### 5.4. Fitts’ Law Modelling

In previous Fitts’ law research, where the interaction may include hand tremor, Welford et al. [57] suggested using a correction to target width that cancels out uncontrolled movement or noise, noting that the effect “might be due to the muscular action of a large member such as the arm not being very finely graded, or to its movement being liable to slight disturbance by factors such as the pulse” [57] (p. 8). The so-called “Welford correction” is an adjustment to target width in calculating the task index of difficulty:

(5) $$ID_{c}\;=\;\log_{2}(\frac{A}{W-c}+1).$$

Welford et al. note that “the constant c might perhaps be attributed to tremor which would increase the scatters and mean that, in order to attain a given level of accuracy, the subject would have to aim at a correspondingly higher level [of accuracy]” [57] (p. 8).

**Figure 7:**
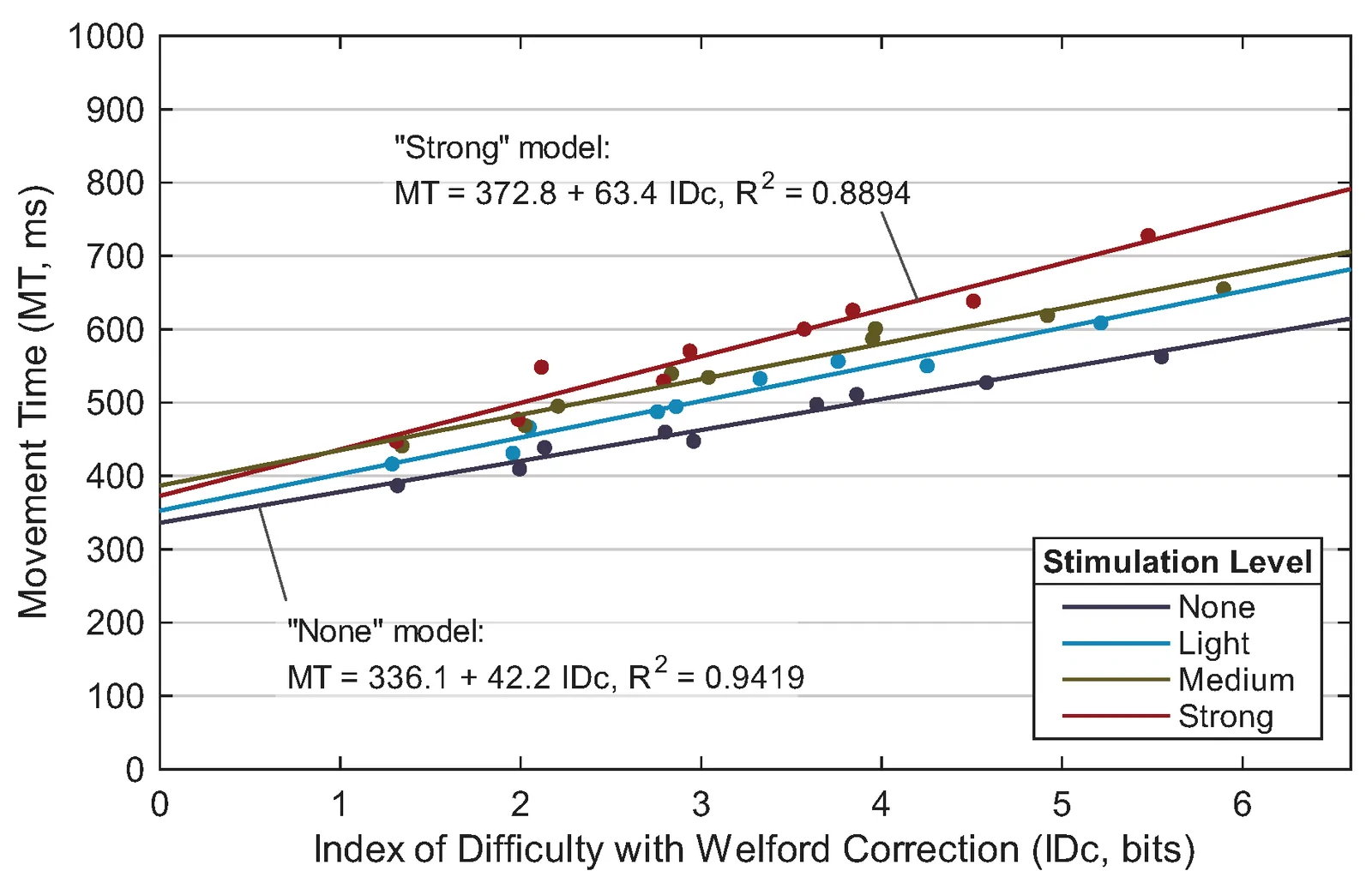
Fitts’ law models for each stimulation level using the Welford correction for uncontrolled movement in the input channel. See text for discussion.

We applied the correction by building Fitts’ law models using Equation (5) for different values of *c*. The models with the highest squared correlations are shown in Figure 7 for each stimulation level. Values of *c* ranged from 65 to 74 pixels, with squared correlations ranging from $R^{2}$ = 0.9419 for no stimulated tremor to $R^{2}$ = 0.8894 for the strong level. The intercepts are all positive but less than 400 ms, which is considered acceptable [42] (p. 758). Note that a higher regression line slope for the strong model corresponds to lower throughput.

Although not shown, the models built without the Welford correction had lower squared correlations, ranging from $R^{2}$ = 0.7391 to $R^{2}$ = 0.8164. Thus, the Welford correction seems appropriate for Fitts’ law models when the interaction involves uncontrolled movement in the input channel: for example, due to hand tremor.

## 6. Touch Correction Prediction

**Figure 8:**
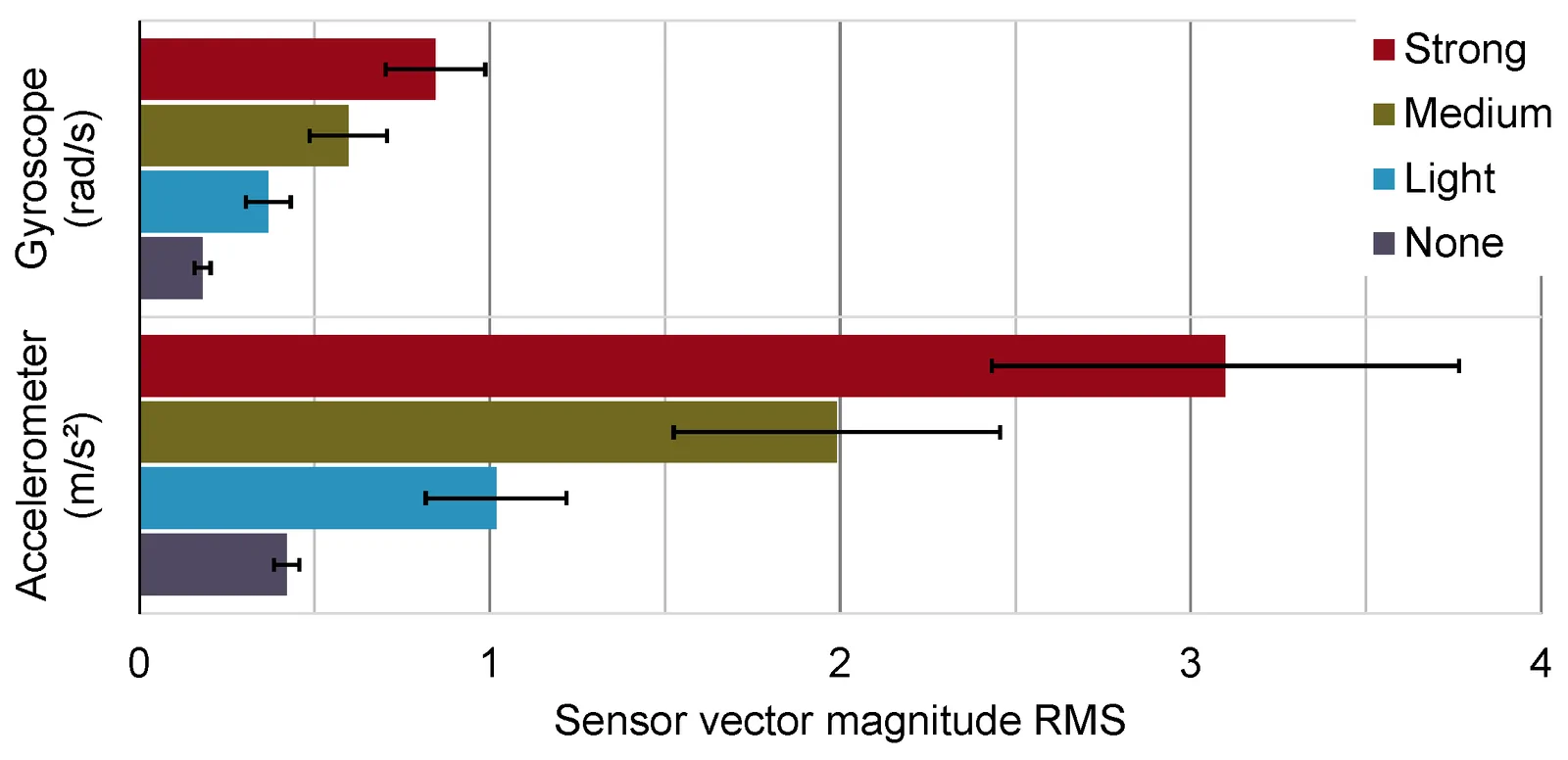
RMS values for accelerometer and gyroscope vector magnitudes per level of TENS activation (error bars show 95% CI).

While recording tap data, the modified FittsTouch application also captured data from the device’s accelerometer and gyroscope. To understand these data, we initially analysed the mean vector magnitude for the accelerometer and gyroscope data (accelerometer data were measured as reported by Android excluding gravity). See Figure 8. An ANOVA indicated a significant difference in the measured acceleration magnitude, or strength, by TENS stimulation level (*F*(1.16, 26.68) = 64.07, *p* < 0.001) with m/s^2^ means of 0.42 (none), 1.02 (light), 1.99 (medium), and 3.1 (strong). Mauchly’s test of sphericity indicated that the assumption of sphericity was violated ($\chi^{2}$(5) = 76.85, *p* < 0.001) and therefore, a Greenhouse–Geisser correction was used (ε = 0.387). Post hoc paired *t*-tests using the Bonferroni correction (α = 0.0083) indicated that the means of all pairs were significantly different.

Similarly, an ANOVA indicated a significant difference in gyroscope data by TENS stimulation level (*F*(1.47, 33.85) = 72.37, *p* < 0.001) with rad/s means of 0.18 (none), 0.37 (light), 0.60 (medium), and 0.84 (strong) (again using the Greenhouse–Geisser correction, ε = 0.491). Post hoc tests indicated that the means of all pairs were significantly different. Interestingly, when excluding the none stimulation level, the recorded accelerometer and gyroscope movement showed a strong linear correlation with applied current.

**Figure 9:**
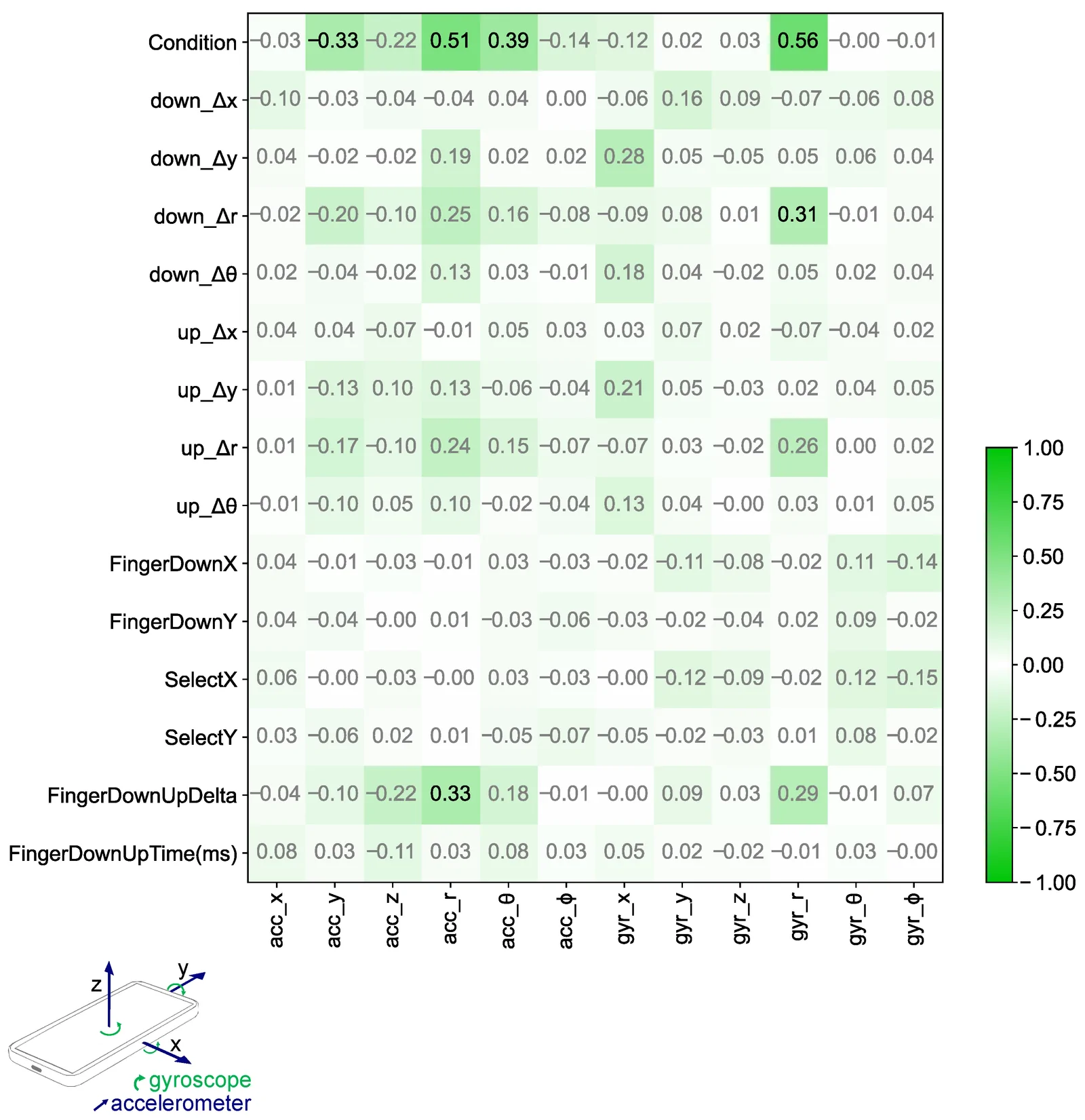
Correlation plot of touch variables vs. sensor variables (with graphical key; colour represents absolute correlation strength).

To visualise potential correlations, we calculated correlations between the touch data and sensor data. Figure 9 shows moderate to strong correlations for accelerometer *y*-axis, accelerometer radius, accelerometer angle, and gyroscope radius, confirming that the strength of induced tremor affects the sensor readings. Note as well the moderate correlations between the size of error on the touch down (Δr) and scale of gyroscope readings (gyr_r) and between finger slippage (FingerDownUpDelta) and the radius of the accelerometer readings.

**Figure 10:**
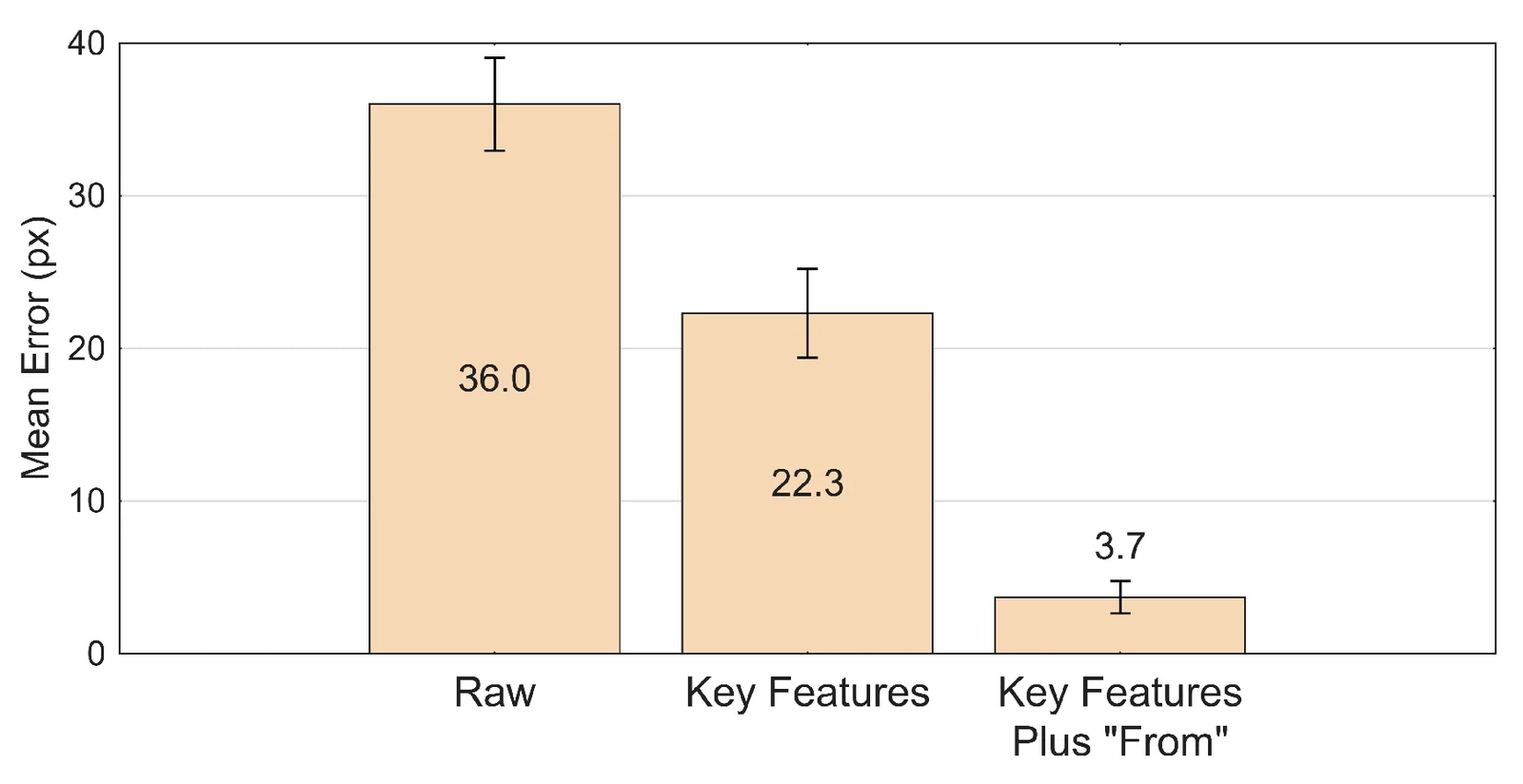
Errors on touches as performed in the current study (left), adjusted using touch and sensor data (middle), and adding in the previous tap location (right). Error bars indicate 95% CI.

The source data for our model were current touch (FingerDownX, FingerDownY, SelectX, SelectY, and FingerDownUpDelta) along with accelerometer and gyroscope data identified from the correlation plot and experimentation (acc_*r*, acc_θ, acc_*y*, and gyr_*r*). For each tap, our aim was to predict the error on finger up (up_Δx, up_Δy). To avoid overfitting, we trained individual models for each participant based on the other participants’ data and did not carry forward data from correlation analysis. We employed a multi-output regression model based on extreme gradient boosting (XGBoost with learning rate = 0.025, n estimators = 1000, max depth = 8, subsample = 0.8, and randomseed = 42, using separate regressors for each target variable and the GPU accelerated histogram tree method). The Euclidean distance was then calculated for original tap error ($|Select_{x,y}-Target_{x,y}|$) and corrected tap using prediction of the error delta ($|Select_{x,y}-\Delta p_{x,y}-Target_{x,y}|$). Participants had an average error of 36.0 pixels (*SD* = 6.1) on raw tap location data (*n* = 24 with each individual’s errors averaged); this was reduced to 22.3 pixels (*SD* = 5.8) in modelling based on key features from the current touch and accelerometer data. See Figure 10.

The results of paired *t*-tests indicated a significant difference with a large effect size between raw errors and corrected errors (*t*(23) = 17.9, *p* < 0.001, *d* = 3.65). Including the previous tap location (“from”) reduced this further to 3.7 pixels average error (*SD* = 2.1), almost eliminating the error (but at a risk of overfitting to the task situation; see the Discussion below).

## 7. Discussion and Limitations

In designing the study, we used fixed stimulation levels with participants who did not have tremor. The results confirm that our choice of 0 mA, 15 mA, 19 mA, and 23 mA (based on pre-trial tests) was appropriate, as each level was noticeably different and the highest level (23 mA), we felt, approached the limit of acceptability. These levels led to significant throughput differences for the none–medium, none–strong, and light–strong pairs. NASA TLX results showed significant differences between all groups, with physical demand yielding the largest effect. Although the relative difference between categories was small, it is noteworthy that significant differences were observed for temporal demand, as feeling pressure to complete fine-detailed tasks is a known consequence of fatigue, frustration, and stress buildup for people with tremor-related conditions.

Sensor data analyses revealed significant differences in measured effects between all induced tremor levels for both the accelerometer and gyroscope. Excluding the no-stimulation condition, the relationship was close to linear, indicating that increased induced tremor did lead to greater phone movement. However, there was a fairly wide range of induced tremor at each level of stimulation. This reflects a trade-off between consistent stimulation levels and consistent induced effects (e.g., as measured by pre-study tremor tests [13]). Although we observed significant differences between conditions and encouraged breaks after TLX completion, some participants reported carry-over effects. When using induced tremor, more research is needed to understand the effect of physiological factors such as fatigue, anxiety, and even caffeine consumption and how pre-study tremor tests impact the main study and on timing between consecutive tests. Overall, our results confirm that the stimulation levels were appropriate, that the models fit well, and that correction is possible. This enables ethically robust future work with people living with tremor, using pre-study tests to assess tremor levels.

Testing was conducted in a private location with the participant standing. While we investigated different stimulation levels and their effects on throughput, movement time, and errors, the study used stimulation on the non-dominant, phone-holding hand only. Future work is needed to combine these results with the single stimulation level for both hands, as reported in Plaumann et al. [12]. We showed that single-hand stimulated tremor could be meaningfully corrected in post-study analyses, while Plaumann et al. required additional sensors for in-study correction when both hands had induced tremor—in future work, we will implement our correction and investigate its use in one- and two-handed induced tremor, as well as in people living with tremor. Our study did not control for precise grip posture, beyond requiring participants to stand while holding the phone in their non-dominant hand and tapping with their dominant hand. The effect of different grips also warrants further investigation.

Fitts’ law modelling showed significant differences in throughput between stimulation levels and, using standardised ISO testing, we quantified the drop in performance for users experiencing tremor while interacting with touchscreens—with drops in performance of 9.4%, 20.3% and 28.9% over three levels of stimulation when compared to performance without induced tremor. The analysis showed significant differences for movement times between levels of stimulation but more dramatic differences in error rate as the levels increased. Index of difficulty modelling showed a good fit with Fitts’ law models (with *R*^2^ between 0.74 and 0.82). Using the Welford correction to adjust for tremor [57], a closer fit was found (*R*^2^ from 0.89 to 0.94). This fit improvement is similar to the improvement found by Ling et al. [7] using Fitts’ Law, but using the Welford correction in ISO standard Fitts’ Law tasks. Our $R^{2}$ values are, however, consistently lower than in their studies, which encourages further investigation of the impact of task difference and experimental conditions, and a full analysis of the different variants of Fitts’ law.

Inspired by others’ success identifying tremor using machine learning (e.g., [7]), we also examined if sensor data could predict the errors while tapping the screen along the *x*-axis and *y*-axis, and thus allow for correction. Correlations did not show a strong relationship between touch location data and sensor data; however, correlations were weak between touch errors (up_Δx and up_Δy) and sensor data (most noticeably gyro_x, acc_r, acc_y, and acc_z). Using extreme gradient-boosting prediction for each user based on other users’ data, we achieved a reduction in each person’s average error from 36 pixels to 22.3 pixels by using data for the current touch and sensor values. This was a significant effect that would improve tap accuracy if implemented. For context, Material Design recommends “making touch targets at least 48 × 48 dp (density-independent pixels)” [58], which translates to approximately 125 pixels on the Pixel 7 used in our studies. This confirms that despite the induced tremor, participants tapped with reasonable accuracy and that we reduced their errors from 29% of a recommended minimum touch target size to 18%. While there is a significant effect, further study is needed to learn if the improved accuracy benefits users in more realistic settings. When the previous touch-point location was added, the model improved considerably to under 4 pixels error (or 3% of the recommended minimum touch-point size). However, the ISO Standard FittsTouch application followed a regular pattern of touches progressing around a circle, as shown in Figure 2. This regular pattern may excessively influence machine learning; this is encouraging and warrants further study to isolate the prediction benefit from the regular touch pattern in the study and to assess it alongside language models for touch-screen typing where the previous touch location is a fairly strong, but imperfect, predictor of the next tap location candidates.

Slippage between finger down and release was highlighted in previous work as being correlated to tremor in the non-dominant hand [29]. Our correlations confirm that slippage (*FingerDownUpDelta*) changes with accelerometer and gyroscope radii. However, it is worth noting that slippage occurred in only 2669 of 14,688 total taps used for learning, with a median slippage of only 12 pixels. This may be a consequence of the task or of only stimulating the phone-holding hand. Further investigation is needed.

Our prediction model excluded touch data from the predicted user to avoid overfitting; the model for each participant was based exclusively on input from others in the study. However, as touch error is personal (e.g., [9]), further research is needed to build models that adapt to the individual and possibly adapt to the level of tremor throughout the day. In the FittsTouch scenario, we could also use the target location to build our correction models. While we did not use the target locations in prediction, they are needed for learning; thus, models of dynamic learning need to be investigated for this context. The FittsTouch application was also prone to unintentional repetitive touches, an issue highlighted by some participants and in the literature (e.g., [30])—identifying and discounting these would further improve accuracy.

## 8. Conclusions and Future Work

This paper presents the first study to quantify the impact of different induced-tremor levels. It also contributes to the design of such studies and confirms that point-select interaction adheres to Fitts’ law—particularly when the Welford correction is applied. Using standardised ISO testing, we observed significantly decreased throughput as tremor increased. This resulted from both decreased movement time and increased errors (no correction was applied during the study). Post hoc analysis of correlations identified gyroscope rotation around *x*, scale of acceleration, and acceleration in *y* and *z* axes as sources for correcting touch errors. Machine learning modelling of the data showed that corrections are possible to improve tap accuracy using smartphone sensor alone (albeit only with induced tremor on the holding hand of healthy participants).

While we have provided the first quantification of the impact of different levels of tremor using the standard ISO tasks, the limitations of the study will be addressed in future work to add additional data points to Fitts’ law modelling for dual induced tremor (both synchronised and non-synchronised) and assess if smartphone-only sensors can still compensate. As our results indicate strong potential, we also plan to work with people living with tremor to better understand how different forms of tremor affect throughput and accuracy in interaction and build machine learning models that are personalised, adaptive throughout the day, and suitable for mobile deployment.

## Data Availability

FittsTouch and sensor data from the study are available at https://doi.org/10.15129/57ebc225-4a0f-406a-b85a-4d7e2011b679 (accessed 1 September 2026).
